# TMT-B after two online mindfulness-based group interventions: Acceptance and commitment therapy and a mindfulness-based emotional regulation intervention in anxiety disorders. Preliminary results

**DOI:** 10.1192/j.eurpsy.2021.2083

**Published:** 2021-08-13

**Authors:** I. Torrea-Araiz, E. Fernández-Jiménez, G. Navarro-Oliver, E. Vidal-Bermejo, T. Castellanos-Villaverde, A. Hospital-Moreno

**Affiliations:** 1 Department Of Psychiatry, Clinical Psychology And Mental Health, La Paz University Hospital, Madrid, Spain; 2 Idipaz, Department Of Psychiatry, Clinical Psychology And Mental Health, La Paz University Hospital, Madrid, Spain

**Keywords:** acceptance and commitment therapy, cognitive flexibility, Mindfulness-based Emotional Regulation, Online treatments

## Abstract

**Introduction:**

There are no studies which address the relationship between mindfulness and cognitive flexibility in interventions carried out online. This is the first study to examine the effect of two online mindfulness-based interventions on this cognitive function.

**Objectives:**

To assess changes on cognitive flexibility after two online mindfulness-based group interventions in adult patients with anxiety disorders.

**Methods:**

This study was carried out in a Mental Health Unit in Spain (Colmenar Viejo, Madrid). Thirteen adult patients (age mean = 51.69 years, ranging from 33 to 69 years, S.D. = 11.56) with anxiety disorders completed the interventions. The group treatments were Acceptance and Commitment Therapy and a Mindfulness-based Emotional Regulation intervention, during 8 weeks, guided by two Clinical Psychology residents. Both interventions were carried out online. The dependent variable was the score on the TMT-B (seconds). A comparison of paired-means was conducted. Statistical significance was set at p < .05.

**Results:**

The normality assumption was met. Statistical power observed = 70.0%. The paired t-test showed statistically significant change between pre-treatment and post-treatment (p = 0.019; Cohen’s d = 0.75), indicating improvement on cognitive flexibility.

**Conclusions:**

These results show a statistically significant and medium/large effect-size change in cognitive flexibility after the two online interventions based on mindfulness. A larger sample size is required to confirm these results. Moreover, other studies need to examine the reliable change on this neuropsychological outcome.

**Disclosure:**

No significant relationships.

